# Shear wave elastography for assessing rectus femoris stiffness: a study on the interaction and reliability of body position and contraction state

**DOI:** 10.3389/fbioe.2026.1768039

**Published:** 2026-01-28

**Authors:** Yating Wang, Tianqi Wang, Xuemei Liu, Xiaona Liu, Ling Li, Huiyu Ge

**Affiliations:** 1 Department of Ultrasound, Beijing Chaoyang Hospital, Capital Medical University, Beijing, China; 2 Department of Ultrasound, Beijing Hospital of Traditional Chinese Medicine, Capital Medical University, Beijing, China

**Keywords:** body position, contraction state, rectus femoris, shear wave elastography (SWE), shear wave velocity (SWV)

## Abstract

**Objective:**

To employ shear wave elastography (SWE) to assess changes in stiffness and reliability in the vastus rectus muscle of healthy adults across different posture-contraction states. We analyzed the main effects and interactions of posture and contraction intensity on shear wave velocity (SWV) and explored its relationship with relevant biological parameters.

**Methods:**

We recruited healthy participants, with two observers using SWE technology to measure the SWV of the vastus rectus muscle in the supine (relaxation, plantarflexion, and dorsiflexion) and sitting (relaxation, leg elevated, and 5-kg loading) positions. Intra- and interobserver reliabilities were evaluated using the intraclass correlation reliability. Repeated-measures analysis of variance was used to examine the main effects and interactions of posture and contraction state on SWV. Additionally, correlations between the SWV and rectus femoris thickness (RF_thick_), circumference (RF_circ_), cross-sectional area (RF_csa_), and contraction index (RF_ci_) were calculated.

**Results:**

Forty-six adults (11 males and 35 females) completed the study. SWE measurements demonstrated high intra- and interobserver reliability across all conditions (ICC >0.80). Two-way repeated-measures analysis of variance revealed significant main effects of body position (F (1,45) = 58.85, *P* < 0.001, η_p_^2^ = 0.567) and contraction state (F (1.76, 78.97) = 104.23, *P* < 0.001, η_p_^2^ = 0.777), with a significant interaction (F (2,44) = 24.66, *P* < 0.001, η_p_^2^ = 0.459). The SWV changes were more pronounced in the 5-kg load position (45.9%). Ultrasound parameters (RF_thick_, RF_circ_, RF_ci_) varied significantly across conditions (*P* < 0.05), whereas the RF_csa_ did not (*P* = 0.194). The SWV was positively correlated with specific architectural parameters, depending on posture, but not with demographic variables (*P* > 0.05).

**Conclusion:**

This study establishes a methodological basis for assessing the stiffness of the RF, identifying posture and contraction as key factors. Our findings underscore the need for standardized measurement and support the future application of SWE in biomechanics, sports, and rehabilitation.

## 1 Introduction

The mechanical properties of skeletal muscles, particularly muscle stiffness, are key indicators of muscle contraction function and strength status. Muscle stiffness evaluation is important in both rehabilitation medicine and sports science (Shinohara et al., 2010; Yoshitake et al., 2014). Handheld dynamometers, isokinetic/isotonic strength testing systems, and surface electromyography are commonly used to assess muscle function. However, these methods either rely on the patient’s subjective cooperation or can only provide indirect mechanical parameters, making it difficult to quantify muscle stiffness directly. Although magnetic resonance elastography can provide non-invasive stiffness measurements, its high cost, limited examination postures, and inapplicability to populations with implanted metal devices restrict its widespread use (Bensamoun et al., 2007; Debernard et al., 2011; Kennedy et al., 2020).

Shear wave elastography (SWE) is an ultrasound technique that was developed in the early 2010s for the visualization and measurement of tissue stiffness (Taljanovic et al., 2017; Klauser et al., 2014). The ultrasonic sound beam automatically induces acoustic radiation, pushing the tissue to produce mechanical vibrations, generating shear waves that propagate throughout the tissue. The propagation velocity of the shear wave in tissue can be accurately quantified and calculated as follows: E = 3ρC^2^ [where E is the elasticity of tissue, ρ denotes the density of tissue, and C is the shear wave velocity (SWV)]; the E value, known as Young’s modulus, represents the mechanical properties of the tissue (Lin et al., 2021; Ličen and Kozinc, 2022; Miura and Miyamoto, 2025). However, this model assumes that the tissue is isotropic and homogeneous, whereas the muscle tissue is inherently anisotropic (Bamber et al., 2013; Ferraioli et al., 2022a; Ferraioli et al., 2022b). Studies have shown that directly using raw SWV values provides a more accurate representation of a muscle’s mechanical properties than the derived Young’s modulus (Zhu et al., 2024; Harmon et al., 2019; Tang et al., 2020). Therefore, in this study, SWV was chosen as the quantitative parameter for assessing rectus femoris stiffness.

The quadriceps femoris is a key muscle group involved in human movement that plays a critical role in maintaining knee stability and performing daily activities. Deterioration in quadriceps function often leads to the loss of knee stability and limited movement capabilities, which are significant factors that affect the quality of life and clinical prognosis of patients with chronic diseases (Morino et al., 2017; Nishiyama et al., 2007). The rectus femoris is the most superficial muscle of the quadriceps and the only muscle that spans both the hip and knee joints and directly influences lower limb performance (Kojic et al., 2022). Existing evidence indicates that myofascial chains can transmit tension between adjacent tissues; during knee extension in the supine position, movement of the ankle joint can likewise propagate stress proximally to the thigh muscles through these myofascial pathways (Mohr et al., 2023; Krause et al., 2016; Liu et al., 2020). In the seated position, knee extension passively stretches the rectus femoris, resulting in an increase in its stiffness (Ličen and Kozinc, 2022; Miura and Miyamoto, 2025). However, to date, no studies have applied SWE under a supine position to systematically evaluate the effects of knee extension combined with ankle joint movement on rectus femoris stiffness. Studies on the rectus femoris SWV in healthy populations are often limited to single-joint positions and fail to systematically simulate the multistate contraction patterns observed in daily activities, making it difficult to comprehensively reveal the biomechanical principles of stiffness changes (Deng et al., 2022).

Therefore, in this study, we aimed to use SWE to systematically assess changes in rectus femoris stiffness in healthy adults across different body positions and contraction states. In this study, we analyzed the main and interaction effects of body position and contraction intensity on SWV, and evaluated the interobserver reliability of SWE measurements. Muscle stiffness is readily influenced by demographic factors such as age, sex, and height, while muscle morphological characteristics (for example, thickness and cross-sectional area) may represent potential confounding variables affecting shear wave velocity measurements. Accordingly, the present study will also examine the associations between shear wave velocity and demographic characteristics as well as two-dimensional ultrasonographic parameters (Gutiu et al., 2025). In this study, we aimed to provide a methodological foundation and clinical reference for the dynamic, noninvasive evaluation of muscle function.

## 2 Materials and methods

### 2.1 Research subjects

This study included 46 healthy volunteers recruited from the ultrasound outpatient department of our hospital in November 2024. The participants included 15 males and 31 females, with an age range of 21–45 years and a mean age of (25 ± 7) years. The exclusion criteria were as follows: (1) physical dysfunction affecting the musculoskeletal system; (2) history of lower limb trauma or surgery; (3) professional athletes; and (4) non-professional individuals engaged in regular high-intensity physical activity or labor (defined as > 5 h per week for >6 months). All participants were recommended to abstain from strenuous exercise for 24 h before the examination. The age, sex, height, weight, body mass index (BMI), and dominant leg information of the participants were recorded. The fundamental information is presented in Table 1. All participants provided written informed consent. This study was approved by the Research Ethics Committee of the Beijing Hospital of Traditional Chinese Medicine, Capital Medical University, and was conducted in strict accordance with the Declaration of Helsinki.

**TABLE 1:** ParticipantCharacteristics (n = 46).

Characteristics	Descriptive statistics
Age (years)	21.0 (21.0,30.0)^a^
Sex (men/women)	11/35
Height (cm)	167.91 ± 8.26^b^
Weight (kg)	60.48 ± 9.86^b^
BMI, kg/m^2^	21.32 ± 1.96^b^

^a^ Values are represented as median (interquartile range).

^b^ Values are represented as mean ± standard deviation (SD).

Abbreviation: BMI, body mass index.

### 2.2 SWE examination

**FIGURE 1:**
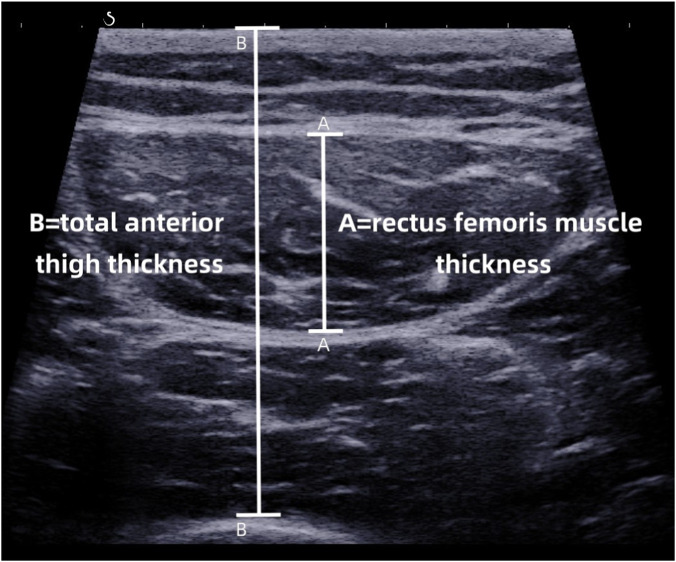
Ultrasound scan with measurements of the dominant thigh. A = rectus femoris muscle thickness; B = total anterior thigh thickness. Rectus femoris contraction index (RFci) = A/B.

**FIGURE 2:**
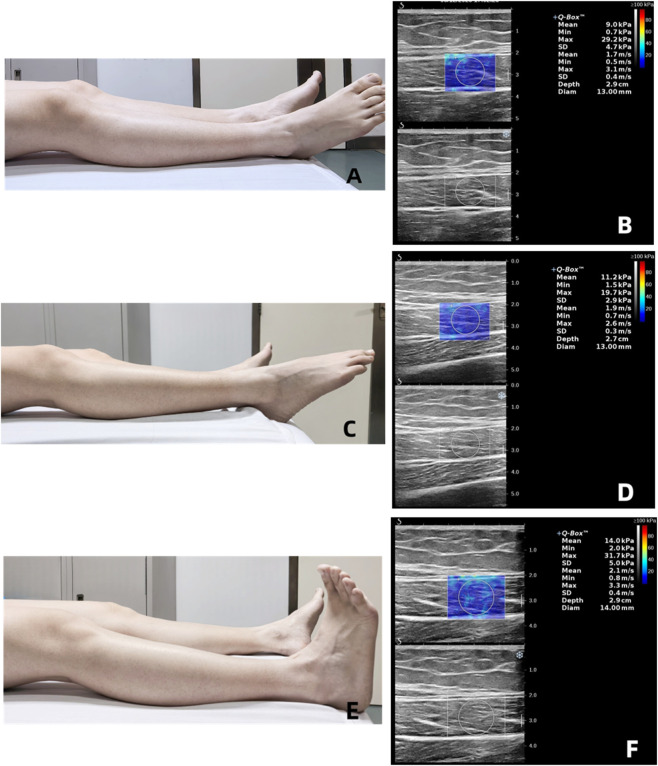
Ultrasound Shear Wave Velocity (SWV) Measurements of the Rectus Femoris in Supine Position under Different Contraction States Subjects were in a supine position. The mean SWV increased from 1.7 to 1.9 m/s to 2.1 m/s in the relaxation **(A,B)**, plantarflexion **(C,D)** and dorsiflexion **(E,F)**.

Ultrasound was performed using an Aixplorer® ultrasound system with a 4–15 MHz linear transducer (Supersonic Imaging, Aix-en-Provence, France). All measurements were acquired in musculoskeletal SWE mode. Before the examination, the participants rested in a temperature-controlled room (24 °C–26 °C) for 15 min to allow leg muscle relaxation. Participants removed their trousers and followed the positional instructions during scanning. The dominant leg was determined by asking the participants which leg they used most often when kicking a ball. The probe was placed lightly at approximately 3/5 of the distance from the anterior superior iliac spine to the upper edge of the patella of the dominant leg, with care taken to avoid applying pressure that may cause muscle deformation due to contact with the probe. Initially, the rectus femoris was identified in transverse view using grayscale ultrasound. The muscle thickness (RFthick), circumference (RFcirc), cross-sectional area (RFcsa), and contractile index (RFci) were measured (Figure 1). Subsequently, SWE was performed in the longitudinal view. A square region of interest was set to cover the full muscle depth, and the largest possible Q-Box™ was placed within it. SWV was recorded once the color map within the Q-Box™ stabilized uniformly. This study incorporated two body positions (supine and seated), with three contraction states defined for each position, yielding six distinct position–contraction state combinations. The SWE measurements were performed in a fixed sequence for all participants: supine relaxation, supine plantarflexion, supine dorsiflexion, seated relaxation, seated leg elevated, and seated 5-kg loading. A 1–2 min interval between different body positions and contraction states is recommended to prevent muscle fatigue. Three valid measurements were acquired repeatedly for each position, and their mean value was taken as the final result for that specific state. The details are as follows:

Supine position. (I) Relaxation: The subject will be positioned supine on the examination bed, with the entire body in a state of relaxation. To execute the movement correctly, the lower limbs must first be fully extended, the hip joint must be positioned in a neutral rotational position, and the ankle joint must be positioned in a relaxed neutral functional position, with a dorsiflexion angle of approximately 0° (Figures 2A,B). (II) Plantarflexion: The participants will be instructed to remain in the supine position with their knees straight before actively performing maximum plantar flexion (Figures 2C,D). (III) Dorsiflexion: The participants will be instructed to maintain the supine position with their knees straight before actively performing maximum dorsiflexion (Figures 2E,F).

**FIGURE 3:**
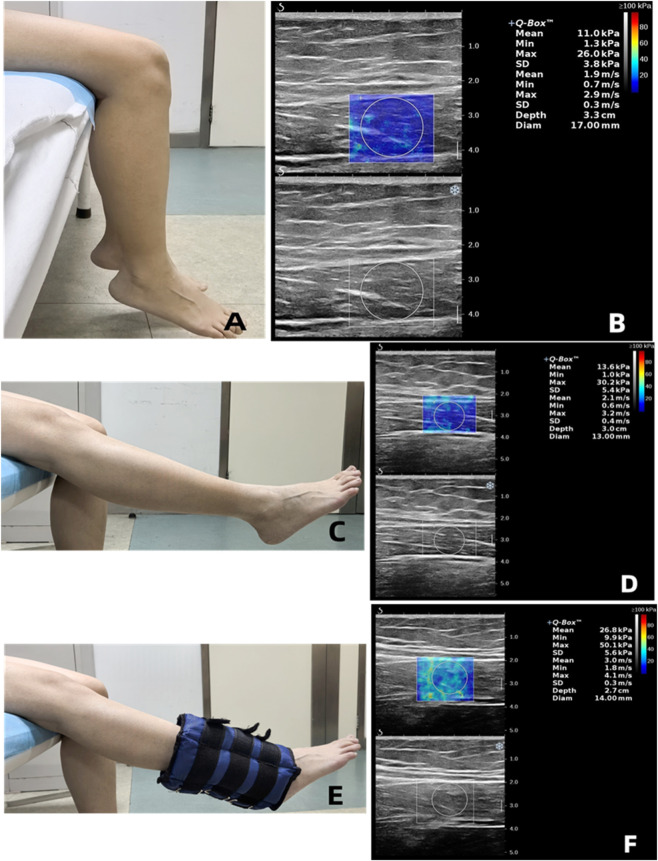
Ultrasound Shear Wave Velocity (SWV) Measurements of the Rectus Femoris in Sitting Position under Different Contraction States. Subjects were in a sitting position. The mean SWV increased from 1.9 to 2.1 m/s to 3.0 m/s in the relaxation **(A,B)**, leg elevated **(C,D)**, and 5-kg loading position **(E,F)**.

Sitting position. (I) Relaxation: The subject will be seated on the examination bed, with their upper and lower limbs positioned at approximately 90°, and their lower limbs positioned in a natural and relaxed state. Participants will be asked to remain relaxed (Figures 3A,B). (II) Leg elevated position: It will be imperative to maintain the non-dominant leg in a relaxed state. The operator then instructs the participant to extend the dominant leg at the knee to maintain a straight position (Figures 3C,D). (III) 5-kg load position: The subject will be required to maintain a state of relaxation in the non-dominant leg, while a 5-kg sandbag will be securely fastened to the ankle of the dominant leg. The operator then provides instructions to the subject, requesting that they actively extend their knee to maintain a straightened leg (Figures 3E,F).

### 2.3 Reliability testing

All subjects underwent a rectus femoris SWE examination by a junior ultrasound physician (with >5 years of experience and having independently completed >100 SWE examinations). Another senior ultrasound physician (with more than 20 years of experience and having independently completed more than 500 SWE examinations) used the same ultrasound instrument to simultaneously conduct independent examinations on 20 participants to assess interobserver reliability.

### 2.4 Statistics

All statistical analyses were performed using SPSS version 29.0. The Shapiro–Wilk test was used to assess the normality of continuous data. Data that followed a normal distribution are presented as the mean ± standard deviation (x̄ ± SD), and comparisons between groups were made using independent samples t-tests. For non-normally distributed data, values are expressed as the median (interquartile range) [M (Q1, Q3)], with the Wilcoxon signed-rank test used to assess between-group differences. Categorical data are presented as frequencies or percentages.

Two-way repeated-measures analysis of variance (ANOVA) was employed to assess the main effects and interaction between body position (two levels) and contraction state (three levels) on the SWV of the rectus femoris. In cases where significant main effects or interactions were detected, *post hoc* pairwise comparisons were performed using the Bonferroni correction method.

The inter- and intra-observer reliability of the measurements was assessed using the intraclass correlation coefficient (ICC) based on a two-way random-effects model for absolute agreement, specifically the ICC(2,k) form where k = 3 represents the number of repeated measurements averaged for each final data point. The following criteria were employed: Scores ranging from 0.40 to 0.75 were designated as poor to fair, while scores of 0.75 and above were categorized as excellent. Pearson or Spearman’s correlation analysis was used to test the correlation between the SWV and demographic and ultrasound parameters. Statistical significance was set at *P* < 0.05.

## 3 Results

### 3.1 Participant characteristics

This study included 46 participants (11 males and 35 females). All participants provided written consent before participation, and Table 1 presents their baseline demographic and health information. The results of the inter- and intra-observer reliability analyses conducted under different body position-contraction states demonstrated that the ICC values of each group exceeded 0.80, indicating that the SWE measurement had adequate reliability (Table 2).

**TABLE 2:** Inter-/Intra-evaluator reliability of SWV measurements.

Variables	Observer 1 (m/s)	Observer 2 (m/s)	SEM (m/s)	Intraobserver ICC(95%CI)	Interobserver ICC(95%CI)
Supine					
Relaxation	1.91 ± 0.21	1.87 ± 0.22	0.08	0.85 (0.719∼0.932)	0.97 (0.883∼0.991)
Plantarflexion	2.03 ± 0.20	1.98 ± 0.19	0.09	0.81 (0.657∼0.914)	0.96 (0.818∼0.986)
Dorsiflexion	2.16 ± 0.26	2.10 ± 0.23	0.11	0.81 (0.651∼0.913)	0.96 (0.761∼0.988)
Sitting					
Relaxation	2.13 ± 0.28	2.08 ± 0.24	0.11	0.83 (0.672∼0.972)	0.97 (0.767∼0.990)
Leg elevated	2.33 ± 0.35	2.25 ± 0.32	0.15	0.81 (0.645∼0.911)	0.95 (0.412∼0.988)
5-kg load	2.64 ± 0.36	2.53 ± 0.32	0.16	0.80 (0.630∼0.905)	0.95 (0.434∼0.988)

SWV: Shear wave velocity.

values are expressed as mean ± SD.

The standard error of measurement (SEM) was calculated based on the intra-observer ICC, and the SD, of Observer 1, using the formula: SEM = SD × √ (1 − ICC).

ICC: intraclass correlation coefficient.

### 3.2 Effects of body position and contraction state on the SWV

**FIGURE 4:**
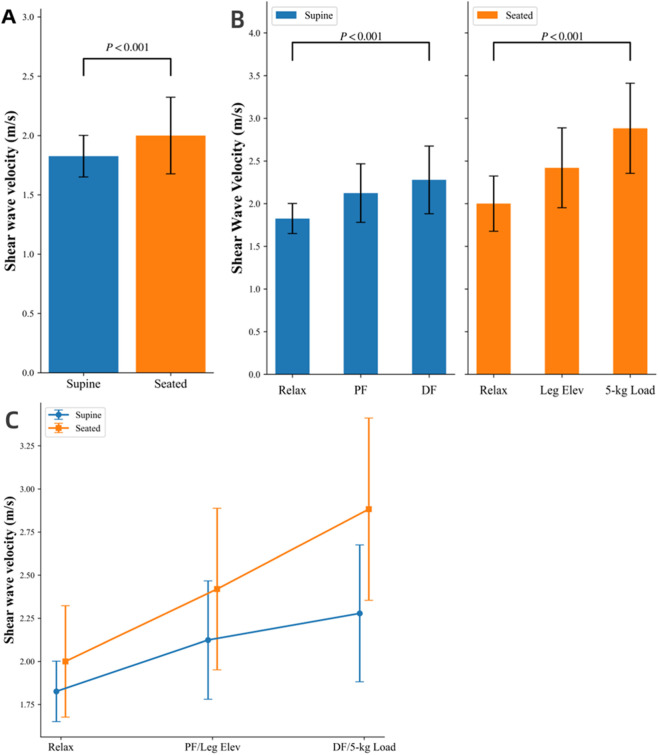
Effect of body position and contraction states on shear wave velocity Relax: Relaxion; PF: Plantarflexion; DF: Dorsiflexion; LE: Leg elevated; 5-kg load: 5-kg load position. **(A)** Comparison of SWV in supine and seated relaxation positions (P = 0.001). **(B)** Comparison of SWV under different contraction states in two body positions (P < 0.001). **(C)** Changes in SWV of the rectus femoris in the three states in supine and sitting positions (P < 0.001). During seated 5-kg loading, SWV increased significantly (2.88 m/s), representing a 22% rise compared with the supine dorsiflexion condition (2.28 m/s).

The two-way repeated-measures ANOVA revealed significant main effects of body position (F (1,45) = 58.85, *P* < 0.001, η_p_^2^ = 0.567) and contraction state (F (1.76,78.97) = 104.23, *P* < 0.001, η_p_^2^ = 0.777), with a significant interaction between the two factors (F (2,44) = 24.66, *P* < 0.001, η_p_^2^ = 0.459). The SWV changes were more pronounced in the 5-kg load position (45.9%). Simple effects analysis showed that (1) the largest change in SWV was observed during the 5-kg load position and dorsiflexion, with the seated position showing a 45.9% increase compared to 25.4% in the supine position (*P* < 0.001); (2) under the supine position, with dorsiflexion resulting in an 8.7% increase compared with plantarflexion (*P* < 0.001); and (3) under the seated position, the effect of contraction on SWV was most prominent, with the 5-kg load position resulting in a 24.6% increase compared with the leg elevated position (*P* < 0.001). These findings suggest that a seated posture combined with a 5-kg load position produces the highest SWV across all conditions (Table 3; Figure 4).

**TABLE 3:** Changes in SWV in different contraction states in the supine and seated positions.

Measures	Supine		Seated	
	ΔPF	ΔDF	Δ leg elev	Δ5-kg load
ΔSWV (m/s)	0.30 ± 0.33	0.45 ± 0.39	0.42 ± 0.32	0.88 ± 0.48
Change (%)	16.7 ± 19.2	25.4 ± 22.4	21.3 ± 16.2	45.9 ± 27.2
Effect size (η_p_^2^)	0.661	0.753	0.794	0.883
95%CI(m/s)	0.110∼0.224	0.187∼0.320	0.324∼0.516	0.740∼1.024

SWV, shear wave velocity; 95%CI, 95% confidence interval.

Δ = Post-Pre, values are expressed as mean ± SD.

Change (%) = POST-PRE/PRE×100.

Δ-PF, change from relaxation to plantarflexion.

Δ-DF, change from relaxation to dorsiflexion.

Δ Leg Elev, change from relaxation to leg elevated position.

Δ5-kg Load, change from relaxation to 5-kg load position.

### 3.3 Ultrasound morphological parameters

Several morphological parameters of the rectus femoris exhibited significant changes across body positions and contraction states (Table 4). The RF_thick_ differed significantly across conditions (F = 16.586, *P* < 0.001). Bonferroni-corrected *post hoc* analyses demonstrated that RF_thick_ in the seated leg elevated position (2.43 ± 0.36 cm) and the seated 5-kg loading (2.54 ± 0.37 cm) was significantly greater than that observed in all three supine positions (relaxed: 1.99 ± 0.27 cm; plantarflexion: 2.08 ± 0.37 cm; dorsiflexion: 2.16 ± 0.32 cm), as well as in the seated relaxation (2.21 ± 0.39 cm; all comparisons *P* < 0.05). Similarly, the RFcirc exhibited a significant main effect (F = 4.692, *P* < 0.001). Post hoc analysis revealed that the RFcircs in the supine relaxation (12.63 ± 1.66 cm) and seated relaxation (12.65 ± 1.43 cm) were significantly greater than that in the seated 5-kg loading position (11.32 ± 1.72 cm; *P* < 0.05). The RFci differed significantly across conditions (F = 2.884, P = 0.015). The RFci was highest in the seated 5-kg loading (0.78 ± 0.07) and was significantly greater in the seated relaxation group than in the supine relaxation (0.73 ± 0.06; *P* < 0.05). The SWV was most strongly influenced by the position–contraction state (F = 41.777, *P* < 0.001). Post hoc tests confirmed that SWV in the seated 5-kg loading (2.88 ± 0.53 m/s) was significantly higher than in all other conditions (*P* < 0.001). In addition, the SWV in the seated leg elevated position (2.42 ± 0.47 m/s) was significantly higher than that in the supine relaxation (1.83 ± 0.18 m/s; *P* < 0.001) and seated relaxation (2.10 ± 0.32 m/s; *P* = 0.021) positions. The SWV in the supine dorsiflexion (2.28 ± 0.40 m/s) was also significantly higher than that in the supine relaxation (*P* < 0.001). In contrast, the RFcsa did not differ significantly across conditions (F = 1.489, *P* = 0.194).

**TABLE 4:** Ultrasound parameters of the rectus femoris muscle in different body positions and contraction states.

Variables	RF_thick_	RF_circ_	RF_csa_	RF_ci_	SWV
Supine					
Relaxation	1.99 ± 0.27^def^	12.63 ± 1.66^f^	8.09 ± 1.99	0.73 ± 0.06^f^	1.83 ± 0.18^bcef^
Plantarflexion	2.08 ± 0.37^ef^	12.06 ± 1.58	7.65 ± 1.79	0.75 ± 0.06	2.12 ± 0.34^aef^
Dorsiflexion	2.16 ± 0.32^ef^	11.86 ± 1.59	7.55 ± 1.86	0.76 ± 0.06	2.28 ± 0.40^adf^
Sitting					
Relaxation	2.21 ± 0.39^aef^	12.65 ± 1.43^f^	8.48 ± 2.00	0.74 ± 0.06	2.10 ± 0.32^acef^
Leg elevated	2.43 ± 0.36^abcd^	11.82 ± 1.66	8.08 ± 2.12	0.76 ± 0.06	2.42 ± 0.47^abdf^
5-kg load	2.54 ± 0.37^abcd^	11.32 ± 1.72^ad^	7.69 ± 2.03	0.78 ± 0.07^a^	2.88 ± 0.53^abcde^
*F*	16.586	4.692	1.489	2.884	41.777
*P*	<0.001	<0.001	0.194	0.015	<0.001

RFthick: Rectus Femoris thickness; RFcirc: Rectus Femoris circumference.

RFcsa: Rectus Femoris cross-sectional aea; RFci: Rectus Femoris contractile index.

SWV: Shear Wave Velocity.

values are expressed as mean ± SD.

Different superscript letters (a-f) within the same column indicate statistically significant differences based on *post hoc* pairwise comparisons with Bonferroni correction. Variables sharing the same letter are not significantly different.

### 3.4 Correlation analysis

The SWV was positively correlated with the RF_ci_ in several body positions, including supine plantar flexion (r = 0.451, *P* = 0.002), supine dorsiflexion (r = 0.604, *P* < 0.001), seated leg elevated (r = 0.312, *P* = 0.035), and seated 5-kg load position (r = 0.451, *P* = 0.002). The SWV was positively correlated with muscle thickness in both supine plantarflexion (r = 0.409, *P* = 0.005) and supine dorsiflexion (r = 0.420, *P* = 0.004). A positive correlation was also observed between SWV and muscle circumference in the supine relaxation state (r = 0.307, *P* = 0.038) and between SWV and muscle area in the supine dorsiflexion state (r = 0.363, *P* = 0.013). Demographic variables (age, sex, height, weight, and BMI) were not significantly correlated with SWV under any condition (*P* > 0.05) (Table 5).

**TABLE 5:** Correlation coefficients between postural conditions of SWV and biomechanical parameters.

Characteristics	SuPine			Seated		
	Relaxation	Plantarflexion	Dorsiflexion	Relaxation	Leg elevated	5-kg load
Age						
r	−0.177	−0.085	0.091	0.153	0.149	−0.043
Sig	0.238	0.575	0.547	0.311	0.322	0.775
Sex, % female						
r	−0.282	−0.055	0.148	0.081	−0.046	−0.049
Sig	0.058	0.719	0.325	0.594	0.763	0.746
Height						
r	−0.158	0.154	0.187	0.005	−0.078	−0.115
Sig	0.295	0.306	0.214	0.972	0.606	0.446
Weight						
r	−0.286	0.048	0.124	−0.040	−0.074	−0.188
Sig	0.054	0.749	0.410	0.790	0.626	0.210
BMI						
r	−0.316	−0.091	0.030	−0.128	−0.072	−0.215
Sig	0.053	0.546	0.843	0.397	0.632	0.152
RF_thick_						
r	−0.010	0.409*	0.420^*^	0.196	0.218	−0.052
Sig	0.946	0.005	0.004	0.192	0.145	0.732
RF_circ_						
r	0.307^*^	0.184	0.167	0.139	0.209	0.232
Sig	0.038	0.220	0.268	0.356	0.164	0.120
RF_csa_						
r	0.235	0.120	0.363^*^	0.231	0.286	0.233
Sig	0.116	0.425	0.013	0.122	0.054	0.119
RF_ci_						
r	0.220	0.451^*^	0.604^*^	0.231	0.312^*^	0.451^*^
Sig	0.142	0.002	<0.001	0.122	0.035	0.002

RF_thick_: Rectus Femoris thickness; RF_circ_: Rectus Femoris circumference.

RF_csa_: Rectus Femoris cross-sectional area; RF_ci_: Rectus Femoris contractile index.

All correlations computed using Pearson’s *r*.

Data met normality assumptions (Shapiro-Wilk P > 0.05).

Bonferroni correction was not applied due to the exploratory nature of analysis.

## 4 Discussion

In this study, we systematically evaluated the SWV measurements of the rectus femoris in healthy participants across different body positions and contraction states, with a focus on analyzing intra- and inter-observer reliability, variation patterns, and correlations with biological parameters. The results demonstrated excellent intra- and inter-observer reliability for SWV measurements across various body positions and contraction states. More importantly, we found a significant interaction between body position and contraction state on SWV, with the highest SWV values observed in the seated 5-kg load position. Furthermore, SWV showed significant correlations with certain biological parameters in specific body position-contraction states. To the best of our knowledge, the current research on rectus femoris stiffness has mainly focused on comparisons before and after training interventions or disease rehabilitation. To the best of our knowledge, this study is the first to systematically explore the SWV variation patterns of the rectus femoris across multiple body positions and contraction states in a healthy population.

Changes in body position and sustained muscle contraction may affect inter- and intra-observer reliability. However, the measurement protocol used in this study demonstrated excellent reliability. We marked the measurement sites on the skin surface to ensure accurate positioning after changes in the body position. To eliminate the effects of stress-induced contractions from position transitions or sustained muscle activation, the participants were instructed to rest for 1–2 min after each position change before measurements were taken. To minimize the measurement error, each body position-contraction state was measured three times, and the average value was used. Notably, our results revealed that the reliability of rectus femoris measurements in the contracted state was slightly lower than that in the resting state. This contrasts with the findings of Young et al., who indicated that the reliability of the cervical multifidus muscle during contraction is higher than that during rest. This discrepancy may be attributed to differences in the anatomical location of the muscle groups (the rectus femoris is a superficial muscle and the multifidus cervicis is a deep cervical muscle) and variations in the SWE operating procedures (Young et al., 2021).

Muscle stretching increases muscle stiffness (Ebihara et al., 2019; Wang et al., 2017; Liu et al., 2021). An important finding of this study is that body position and contraction state have a significant interaction effect on rectus femoris SWV. Specifically, the increase in SWV under seated 5-kg load position was much greater than that in the other states, suggesting that the effect of body position on rectus femoris stiffness depends on the contraction state. The stiffness changes induced by contraction are also influenced by the body position. This finding emphasizes the need for strict standardization of the body position and contraction conditions when using SWV to assess and compare muscle stiffness. Furthermore, resistance training in the seated position may be beneficial for activating and strengthening the rectus femoris. The assessment of rectus femoris SWV in the seated leg elevated position with a 5-kg load may serve as a quantitative indicator of muscle functional recovery in patients with postoperative or neurological impairment.

The body position and contraction state also had significant effects on the SWV of the rectus femoris. This finding aligns with the observations of Alfuraih et al. (2019), who demonstrated that the quadriceps femoris muscle exhibited elevated levels of SWV during passive stretching. Although the rectus femoris is in a relatively elongated anatomical position in the supine position, the muscle can achieve maximum relaxation owing to the full body support provided by the examination table. Conversely, in the seated position, although the rectus femoris is structurally shortened, it must engage in postural tension regulation to resist the gravitational pull of the lower leg and maintain balance, as it spans both the hip and knee joints. Therefore, the supine relaxed position may serve as the preferred posture for establishing individualized baseline muscle stiffness, particularly for monitoring changes in muscle hardness or evaluating the immediate effects of relaxation interventions, such as myofascial release or cryotherapy.

Consistent with previous studies, the SWV of the rectus femoris in this study increased from a relaxed seated position to a contracted state under a 5-kg load. Tang et al. (2020) reported a linear correlation between the rectus femoris SWV and external resistance, with no differences based on age or sex (Tang et al., 2020). Liu et al. confirmed that the SWV of the medial head of the gastrocnemius gradually increases as the ankle joint moves from 0° dorsiflexion to 30° (Liu et al., 2021). Lee et al. also stated that the stiffness in the active contraction state was significantly higher than that in the relaxed state among multiple healthy human muscles (Lee et al., 2021).

However, there were some differences between these studies. Wang et al. (2017) found that the shear modulus of the vastus intermedius was significantly higher at 90° knee flexion than at 60° knee flexion (Wang et al., 2017). This difference may be due to the use of a rehabilitation system to support the lower leg in their study, whereas the participants’ lower legs were in a naturally suspended position in the present study. Additionally, Mohr et al. suggested that during knee extension, ankle dorsiflexion could transmit force from the lower leg to the thigh through extramuscular connective tissues, such as the deep fascia (Mohr et al., 2023). This may explain why the rectus femoris SWV increased during both plantar flexion and dorsiflexion of the ankle in the supine position in the present study.

We found that while muscle thickness is easily influenced by individual factors, such as body weight and height, RFci helps mitigate the confounding effects of subcutaneous fat, providing a better representation of the contraction ratio of the rectus femoris in the anterior thigh. Consistent with the findings of Guerreiro et al., this study also revealed that RFci exhibited a stronger and more pronounced correlation with the rectus femoris SWV under contraction, which more directly reflected the muscle’s reserve strength (Guerreiro et al., 2017). Our study further refines the concept of relative thickness proposed by Agyapong-Badu et al. by extending it from the quadriceps muscle group to specifically focus on the rectus femoris (Agyapong-Badu et al., 2014). Interestingly, the correlation between rectus femoris SWV and thickness disappeared in the seated contraction state. This may be due to the spatial constraints imposed by the surrounding fascial compartments and the integrative mechanical synergy of the surrounding muscles in the seated position (Germain et al., 2020; Germain et al., 2024; Colonna and Casacci, 2024; Germain and Perrin, 2023). Therefore, the SWV of the rectus femoris in the seated contraction states may no longer reflect the mechanical properties of the muscle in isolation but rather the integrated stiffness of the entire knee-extensor muscle group. This approach can be applied to evaluate the stiffness and force reserve of the quadriceps as a functional unit during activities such as running or jumping, providing a basis for precision training.

## 5 Limitations

This study has certain limitations. First, the participants were predominantly young females. In this study, we aimed to explore the variation patterns of the rectus femoris SWV across different body positions and contraction states, which may be less influenced by demographic characteristics. Nevertheless, the relatively homogeneous sex and age distributions of the sample may limit the generalizability of our findings to a broader population. Future research should include a broader age range and a more balanced sex distribution of participants to further validate the universality of rectus femoris SWV variation patterns and uncover potential group differences. Second, we exclusively collected basic biological parameters. The effects of other variables (e.g., physical activity level and muscle mass) on the SWV of the rectus femoris should be further investigated. Finally, this study primarily relied on standardized movement instructions (actively performing maximal plantar). Future work will integrate simultaneous monitoring of surface electromyography signals or joint torque to establish quantitative relationships between muscle contraction and SWV changes more rigorously.

## 6 Conclusion

This study provides a methodological basis for assessing vastus rectus muscle stiffness under different postural and contraction conditions by identifying interactive effects and key influencing factors. This emphasizes the need for strict standardization of posture and contraction conditions when evaluating muscle stiffness. These findings provide a crucial foundation for future applications of SWE in biomechanical modeling, performance evaluation, and rehabilitation monitoring.

## Data Availability

The data analyzed in this study is subject to the following licenses/restrictions: The datasets generated and analyzed during the current study are not publicly available due to restrictions imposed by the ethical approval and informed consent agreements, which state that participant data must be protected to ensure privacy and confidentiality. The data contain sensitive personal health information. However, de-identified data may be made available to qualified researchers upon reasonable request, subject to approval by the Ethics Committee of the Beijing Hospital of Traditional Chinese Medicine, Capital Medical University and the execution of a data use agreement. Requests should be directed to the corresponding author at email 1040843414@qq.com. Requests to access these datasets should be directed to HG, 1040843414@qq.com.
